# Are the deficits in navigational abilities present in the Williams syndrome related to deficits in the backward inhibition?

**DOI:** 10.3389/fpsyg.2015.00287

**Published:** 2015-03-18

**Authors:** Francesca Foti, Stefano Sdoia, Deny Menghini, Laura Mandolesi, Stefano Vicari, Fabio Ferlazzo, Laura Petrosini

**Affiliations:** ^1^Department of Psychology, Sapienza University of RomeRome, Italy; ^2^IRCCS Fondazione Santa LuciaRome, Italy; ^3^Child Neuropsychiatry Unit, Department of Neuroscience Bambino Gesù Children’s Hospital RomeItaly; ^4^ Department of Motor Science and Wellness, University of Naples “Parthenope”Naples, Italy

**Keywords:** visuospatial task-switching, verbal task-switching, executive function, spatial ability, spatial navigation

## Abstract

Williams syndrome (WS) is associated with a distinct profile of relatively proficient skills within the verbal domain compared to the severe impairment of visuo-spatial processing. Abnormalities in executive functions and deficits in planning ability and spatial working memory have been described. However, to date little is known about the influence of executive function deficits on navigational abilities in WS. This study aimed at analyzing in WS individuals a specific executive function, the backward inhibition (BI) that allows individuals to flexibly adapt to continuously changing environments. A group of WS individuals and a mental age- and gender-matched group of typically developing children were subjected to three task-switching experiments requiring visuospatial or verbal material to be processed. Results showed that WS individuals exhibited clear BI deficits during visuospatial task-switching paradigms and normal BI effect during verbal task-switching paradigm. Overall, the present results suggest that the BI involvement in updating environment representations during navigation may influence WS navigational abilities.

## Introduction

Williams syndrome (WS) is a relatively rare genetically based neurodevelopmental disorder with estimates of prevalence between 1 in 7500 and 1 in 20,000 births (Stromme et al., 2002). The disorder is caused by a *de novo* microdeletion on the long arm of chromosome 7, specifically 7q11.23 (Ewart et al., 1993). The disorder has attracted a great deal of interest from cognitive neuroscientists due to a unique profile associated with a behavioral and cognitive phenotype (Martens et al., 2008). Namely, behavioral phenotype of WS individuals is associated with inattention, distractibility, and hyperactivity alongside social disinhibition and non-social anxiety (Leyfer et al., 2006; Rhodes et al., 2011), while their cognitive phenotype has relative strength points in verbal abilities and face recognition and weakness points in visuospatial processing (Vicari et al., 2005, 2006; Brock, 2007; Hocking et al., 2008; Karmiloff-Smith, 2012).

Visuospatial difficulties of WS individuals have been well documented both in small- and large-scale tasks (Bellugi et al., 2000; Hoffman et al., 2003; Farran and Jarrold, 2004;Mandolesi et al., 2009; Farran et al., 2010, 2012; Foti et al., 2011). WS individuals exhibit specific difficulties on tasks requiring the encoding of spatial relationships between landmarks in a small-scale array (Nardini et al., 2008; Bernardino et al., 2013). In addition, difficulties on tasks requiring imagined rotations of the self and objects have been described in WS individuals, indication of their difficulties with encoding spatial locations of objects in relation to the self and other objects (Farran et al., 2001; Stinton et al., 2008). Notably, the structural and functional hippocampal abnormalities described in WS individuals are retained to be associated with their difficulties on tasks that require the ability to update egocentric spatial locations within an allocentric frame of reference (Meyer-Lindenberg et al., 2005). Moreover, the ability to imagine the self rotating predicts performance on navigation tasks that require the individual to constantly update self-to-object and object-object locations when moving through an environment (allocentric spatial coding; Kozhevnikov et al., 2006; Broadbent et al., 2014). Consequently, the WS difficulties on small-scale tasks match up their difficulties in large-scale tasks, particularly the tasks requiring allocentric encoding. On large-scale spatial tasks, WS individuals are able to learn to navigate and retrace a route both in real-world and virtual environment although they show deficits in understanding spatial relationships in the environment (Farran et al., 2010, 2012). It is known that the ability to learn a route precedes (developmentally and temporally) the ability to encode environmental spatial relationships (Siegel and White, 1975). Thus, as advanced by Farran et al. (2010), the poor WS learning of environmental relationships suggests that WS individuals are able to learn a novel route by relying on a specific set of turns and landmarks but they would not be able to deviate from that route to find a short cut or to make a detour. In a large-scale radial arm maze (RAM), WS individuals display impaired procedural competencies, spatial working memory deficits, and perseverative tendencies revealing explorative and mnesic deficits and severe problems in cognitive planning (Mandolesi et al., 2009). In another large-scale study with multiple rewards, WS individuals displayed disorganized and ineffective search strategies as well as a deficient understanding of the environmental layout (Foti et al., 2011). These findings are in line with the results of a recent study examining the navigational strategies spontaneously employed by WS individuals in a large-scale virtual environment task as well as their ability to use allocentric strategies (Broadbent et al., 2014). During spontaneous navigation, the WS individuals did not employ any sequential egocentric strategy and followed the path until the correct environmental landmarks were found, suggesting their use of a time-consuming and inefficient view-matching strategy for wayfinding. Once more, WS difficulties in determining short-cuts and in developing mental representations of the environmental layout demonstrated their deficits in allocentric spatial coding (Broadbent et al., 2014).

Williams syndrome visuospatial deficits have been at least partially attributed to their impairment in dorsal stream structure and function (dorsal stream deficit hypothesis; Atkinson et al., 2003). However, this hypothesis does not wholly explain the WS visuospatial impairment since deficits in dorsal stream functioning are not specific to this clinical population (Atkinson and Braddick, 2011). The most recent studies consistently advance WS deficits in prefrontal executive control during strategic manipulation of spatial information (Vicari et al., 2006; Menghini et al., 2010; Rhodes et al., 2010, 2011; Costanzo et al., 2013). Executive control is defined as an extensive set of high-order mental operations (including planning, inhibitory control, working memory, attentional flexibility, problem solving) that organize and regulate goal-directed behavior (Miyake and Shah, 1999). The executive control can be fractionated into separable, even if not fully independent, processes as the ability to shift between different mental sets or tasks (*Shifting*), update and monitor working memory representations (*Updating*), and selectively attend to stimuli and inhibiting responses (*Inhibition*; Miyake et al., 2000; Miyake and Friedman, 2012). Interestingly, WS individuals exhibit impaired set-shifting, inhibition and working memory abilities on visuospatial but not verbal tasks (Atkinson et al., 2003; Jarrold et al., 2007; Menghini et al., 2010; Rhodes et al., 2010; Carney et al., 2013; Costanzo et al., 2013). Also in a recent study WS individuals showed inhibition deficits, problems in re-engaging attentional control processes after making an error, and generalized deficits of concentration (Greer et al., 2013). Overall, these findings indicate deficits of specific executive functions in WS.

The ability to switch among different cognitive representations is usually investigated by means of task-switching paradigms, in which people perform one of two or more tasks (rules) with identical stimuli in each experimental trial, with a cue indicating the relevant task. In any trial, the task can be repeated (A–A) or changed (B–A). Switching from one task to another implies a behavioral cost (*switch cost*), as evidenced by the increase in reaction times (RTs). Importantly, switching back to a recently executed task (such as performing the A task as the third trial in an A–B–*A* sequence) is harder than switching back to a less recently executed task (such as performing the A task as the third trial in a C–B–*A* sequence), as evidenced by the slowing of RTs on the third trial in an A–B–A sequence compared to the third trial in a C–B–A sequence (*Backward Inhibition*, BI; Mayr and Keele, 2000; Kiesel et al., 2010; Koch et al., 2010; Vandierendonck et al., 2010; Sdoia and Ferlazzo, 2012). The BI is an inhibitory process hold to suppress the representations of the control settings from the preceding task during intentional task or goal shifts. The assumptions are that inhibition is applied to the preceding task set at each task switch and that the inhibition dissipates slowly (e.g., Dagenbach et al., 2007). Hence, RTs slow and errors increase when the current task had been already executed at the lag-2 trial compared to when it had not been executed (A–B–*A* sequence compared to C–B–*A* sequence). BI process allows suppressing the mental representation (task set) of the just executed task, reducing thus its potential interference on a new task and allowing flexibly adapting to continuously changing contexts and environments (Arbuthnott, 2008; Koch et al., 2010; Sdoia and Ferlazzo, 2012).

To efficiently navigate in complex environments a continuous updating of and shifting among representations (from egocentric to allocentric and vice versa) and strategies (from praxic to cognitive mapping and vice versa) are necessary (Burgess, 2006; Igloi et al., 2009). Switching back and forth among representations and strategies requires executive capacities, as the BI. Intriguingly, we recently advanced the role of the BI in spatial navigation in individuals with developmental topographical disorientation who never developed topographical competencies and show selective difficulties in orienting and way-finding (Iaria et al., 2005, 2009; Bianchini et al., 2010, 2014; Palermo et al., 2014a,b). In the spatial task-switching they failed to shift behavior for adapting their search strategies to the previous outcomes, demonstrating thus a peculiar defect of BI in spatial domain. The proposal we suggested was that in Developmental Topographical Disorientation the updating of the zoomed representations of the just navigated space is malfunctioning, just in relation to a defective BI.

To date little is known about the influence of executive control in general, and of BI in particular, on navigational abilities in typical and atypical development. The present study is aimed at studying the relationship between the BI and navigational abilities in WS individuals. To this aim, spontaneous navigational strategies employed in a large-scale RAM task by a group of WS individuals were compared with those of a mental age- and gender-matched group of typically developing (TD) children. In the same groups, BI performances were assessed by means of small-scale visuospatial and verbal task-switching (VeTS) paradigms. In particular, the ability in switching among spatial representations was examined by means of the visuospatial task-switching (VsTS), while the ability in switching among verbal stimuli not tapping spatial components was examined by means of the VeTS. This procedure allowed analyzing whether the eventual executive deficit of WS individuals was limited to spatial domain or it was part of a more general impairment.

## Materials and Methods

### Participants

Fifteen individuals with WS and 15 mental age- and gender-matched TD children were recruited to participate in the study. All WS individuals (mean chronological age, 19.3 years ± 1.9; nine males) and TD children (mean chronological age, 6.7 years ± 0.3; nine males) were right-handed and native Italian speakers and belonged to upper-middle class families, as assessed by a short questionnaire that was given to the participants’ parents. In all participants, the clinical diagnosis of WS was confirmed by the genetic investigation FISH (fluorescent *in situ* hybridization) demonstrating the characteristic deletion on the chromosome band 7q11.23. In addition to the clinical and genetic diagnosis of WS, selection criteria for study recruitment included normal or corrected-to-normal vision.

The TD children were recruited from local schools, and their parents reported that they were in good health. Exclusion criteria were reports of neurological signs and history of language delay or learning disability.

The participants’ cognitive level was measured using the short version of the Leiter-R intelligence scale (Roid and Miller, 2002). Mean mental age in the WS group was 6.5 years ± 0.2 and in the TD group was 6.6 years ± 0.3, whereas mean intelligence quotient (IQ) was 56.9 ± 2.5 and 105.6 ± 1.7, respectively. Overall, the groups differed in chronological age [*F*_(1,28)_ = 41.8, *p* < 0.00001, $\eta_{p}^{2}$ = 0.59] and IQ [*F*_(1,28)_ = 263.1, *p* < 0.00001, $\eta_{p}^{2}$ = 0.90] but not mental age [*F*_(1,28)_ = 1.33, *p* = 0.26, $\eta_{p}^{2}$ = 0.045]. Moreover, visuo-motor integration (VMI) and memory functions were assessed by VMI (Beery and Buktenica, 2000), visuo-spatial short-term memory (VSS), and visuo-object short-term memory (VOS) tests (Vicari, 2007). Statistical comparisons between groups are reported in **Table 1**.

**Table 1:** Statistical comparisons of performances of Williams syndrome (WS) and typically developing (TD) participants.

Cognitive domain	WS Mean (±SEM)	TD Mean (±SEM)	Group effect *F*_(1,28)_, *p*; $\eta_{p}^{2}$
Visuo-motor integration (VMI)	11.53 (±0.56)	15.47 ( ±0.38)	*F*= 34.03, *p*< 0.00001 $\eta_{p}^{2}$ = 0.55
Visuo-spatial short-term memory (VSS)	2.07 (±0.15)	3.67 (±0.19)	*F*= 43.82, *p*< 0.00001 $\eta_{p}^{2}$ = 0.61
Visuo-object short-term memory (VOS)	2.60 (±0.13)	2.87 (±0.17)	*F*= 1.60, *p* = 0.22 $\eta_{p}^{2}$ = 0.05

Informed consent was obtained from all participants and their families, and the study was conducted according to the Declaration of Helsinki.

## Experiment 1: Visuospatial Task-Switching (VsTS)

**FIGURE 1:**
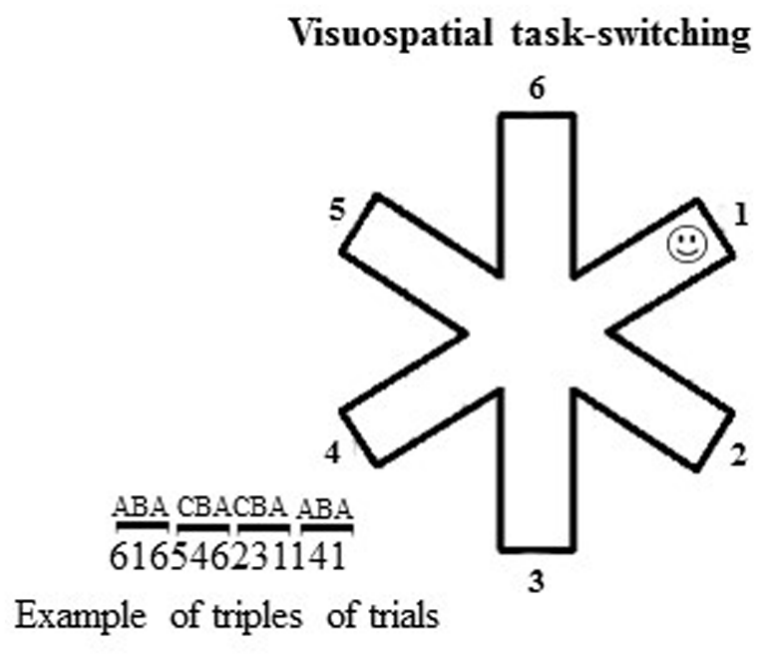
**Schematic representation of the visuospatial task-switching (VsTS) paradigm (Experiment 1).** On the left, an example of triplets of trials (CBA and ABA). The numbers indicate the arms of the 6-arm star.

To analyze the ability in switching among different spatial representations, the participants were tested in a small-scale computerized VsTS (Palermo et al., 2014a) that combined the spatial features of the large-scale RAM (Mandolesi et al., 2009; Foti et al., 2011) and the features of the standard task-switching paradigms (Mayr and Keele, 2000; Sdoia and Ferlazzo, 2012). Specifically, the VsTS paradigm required participants to switch among spatial positions to search for a target (a smiley face) that was hidden at the end of one arm of a 6-arm star on a computer touch screen (**Figure 1**). According to the hypothesis that the BI plays a role in navigating in complex environments by inhibiting the environmental representations once they have been used, we expect that individuals should take longer (that is, they explore more arms) to find the target placed in the same position as two trials before (A–B–*A* sequence) than when placed in a different position (C–B–*A* sequence). It is worth noting that whilst BI effects have been more often observed on RTs to simple tasks, both theory and empirical evidence suggest that they can also be observed on other performance measures, such as the error rates (e.g., Koch et al., 2010).

### Materials and Procedure

At the beginning of each trial, the outline of a 6-arm star appeared at the center of a touch screen on a subtending white background (14.25^∘^width × 14.25^∘^height of visual angle). The target was a small, yellow, stylized representation of a smiley face (smiley; 0.48^∘^width × 0.48^∘^height of visual angle), hidden at the distal location of one of the six arms (**Figure 1**). Participants had to touch the arms successively until they found the smiley. When the smiley was discovered, a new trial (with a new smiley position) began. Each participant performed 180 trials, in which randomized series of 30 non-alternating (CBA) and 30 alternating (ABA) triplets appeared. In the CBA sequences, the smiley was in a different arm, randomly selected, in each trial of the triplet. In the ABA sequences, the smiley was at the same arm, randomly selected, in the first and third trials of the triplet. BI effect occurred when a significantly higher number of arms was touched in the ABA vs. CBA sequences.

#### Parameters

We measured the *number of arms* touched on searching for the smiley in ABA, CBA sequences and both of them; the *number of errors*, re-explorations of the same arm in the same trial; the *perseverations*, exploration of the same arm (e.g., 2–2) or the same string of arms (1–2–1–2) consecutively touched in the same trial; the *starting arm,* the first arm touched in each trial. The percentage of response pairs for which the touched arms were adjacent was also computed as *adjacency values* (e.g., touching arms 2–3, or 6–5, or 6–1 sequentially; **Figure 1**). Lower adjacency values reflect more scattered exploration, whereas higher values indicate more systematic and regular exploration (Towse and Neil, 1998).

### Results and Discussion

**FIGURE 2:**
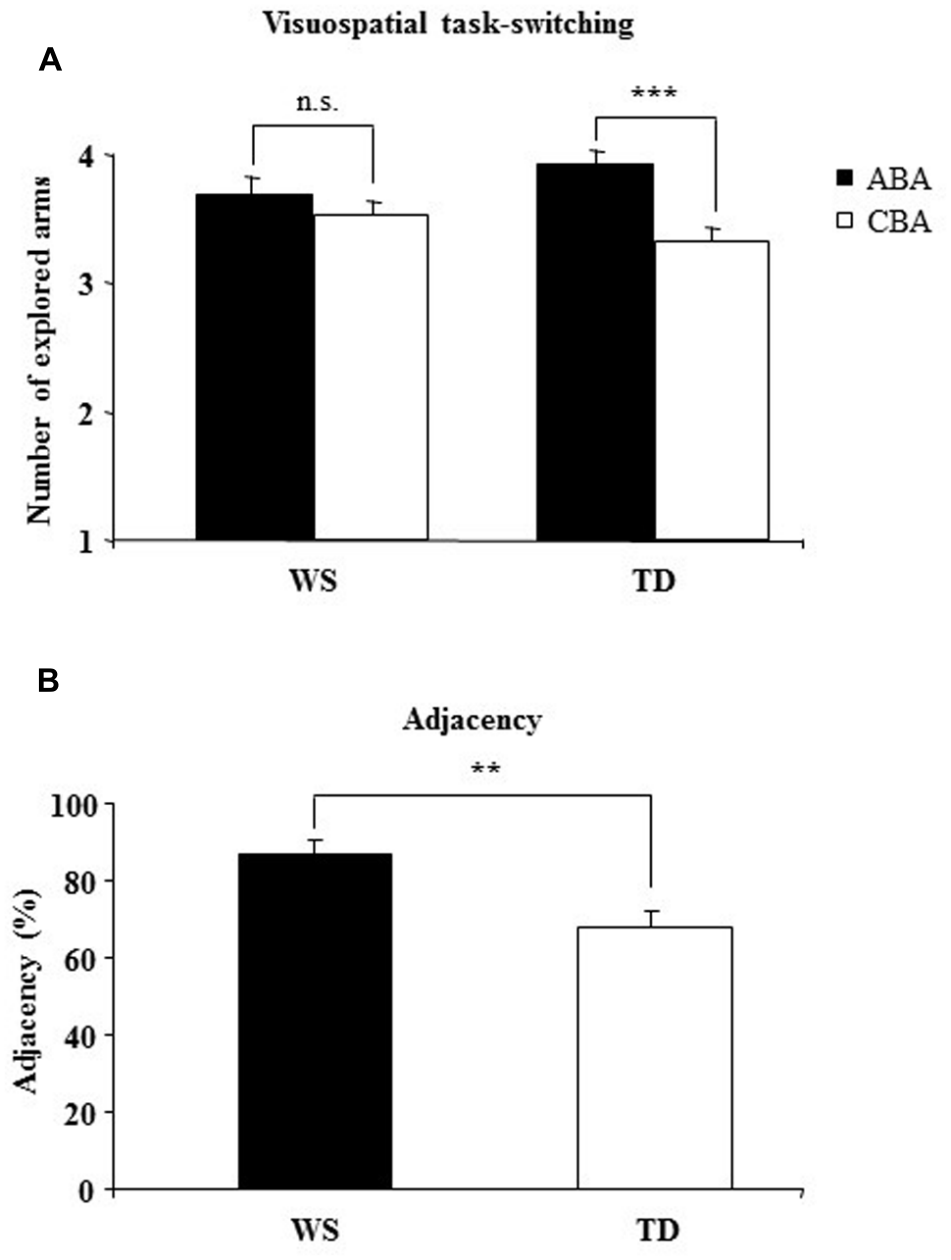
**(A)** Backward inhibition (BI). Number of explored locations on finding the target in alternating (ABA) and non-alternating (CBA) sequences of trials for Williams syndrome (WS) and typically developing (TD) participants. **(B)** adjacency. The percentage of response pairs for which the arms were adjacent for WS and TD participants. Data are expressed as mean ± SEM. The asterisks indicate the significance level of the *post hoc* comparisons between groups **(A)** or one-way ANOVA **(B)** (^∗∗^*p*< 0.005, ^∗∗∗^*p*< 0.0005).

A two-way ANOVA (group × sequence) on the *number of arms* revealed a not significant group effect [*F*_(1,28)_ = 0.004, *p* = 0.94, $\eta_{p}^{2}$ = 0.0002], indicating that when ABA and CBA sequences were considered together both groups of participants touched the same numbers of arms. The sequence effect [*F*_(1,28)_ = 20.60, *p* = 0.0001, $\eta_{p}^{2}$ = 0.42] and the interaction [*F*_(1,28)_ = 7.59, *p* = 0.010, $\eta_{p}^{2}$ = 0.21] were significant. *Post hoc* comparisons on interaction revealed that only TD participants explored more arms on the ABA than CBA sequences (*p* = 0.0002)*,* while WS participants explored the same number of arms in the both sequences (*p* = 0.22), indicating that the visuospatial BI effect was lacking in WS participants (**Figure 2A**).

Notably, no differences between groups were found in *number of errors* [one-way ANOVA: *F*_(1,28)_ = 1.04, *p* = 0.32, $\eta_{p}^{2}$ = 0.036; TD $\overset{-}{x}$ = 11.20 ± 2.70; WS $\overset{-}{x}$ = 14.58 ± 1.92] and *perseverations* [one-way ANOVA: *F*_(1,28)_ = 0.73, *p* = 0.40, $\eta_{p}^{2}$ = 0.025; TD $\overset{-}{x}$ = 5.2 ± 1.70; WS $\overset{-}{x}$ = 7.04 ± 1.44]. As revealed by a one-way ANOVA on the *starting arm* [*F*_(1,28)_ = 6.56, *p* = 0.016, $\eta_{p}^{2}$ = 0.19]*,* WS participants clicked the same starting arm significantly more frequently than the TD children (WS: 46.34%; TD: 29.78%). As revealed by a one-way ANOVA on *adjacency values* [F_(1,28)_ = 10.84, *p* = 0.003, $\eta_{p}^{2}$ = 0.27],WS participants explored adjacent arms significantly more than TD children. These results indicate that in searching for the smiley, WS participants explored the 6-arm star more systematically than TD children and moved primarily from one arm to the adjacent arm (**Figure 2B**).

The main result of the Experiment 1 was that whereas TD participants touched more arms in the ABA vs. CBA sequences, WS individuals did not follow this pattern and exhibited search strategy independent of where the target was found on the previous trials as indicated by their similar number of touched arms in ABA and CBA sequences. In WS individuals, the target location did not become inhibited and hence was equally explored in the successive trial, strong indication of their lack of a visuospatial BI effect. This result implicates that the inhibitory control, mediated by the BI, shapes the strategies that individuals use to explore ever-changing environment efficiently, dismissing previously visited locations. Interestingly, in the search for the target the WS individuals undertook more systematic and regular exploration, as evidenced by their high adjacency values. Such an ordered exploration reduced the spatial working memory load and helped WS individuals to perform the task as successfully as TD children, as indicated by the lack of differences in the number of touched arms, errors, or perseverations. To determine the role of the BI in modulating spontaneous spatial exploration the same groups of participants were tested in a large-scale RAM.

## Experiment 2: Visuospatial Task-Switching in an Ecological Environment

The aim of Experiment 2 was to determinein a large-scale RAM whether previously visited (and thus theoretically inhibited) locations were explored less frequently than never-visited locations. Whether as indicated by the results of Experiment 1, BI favors the exploration of new sites, participants who displayed a BI effect should generate more CBA than ABA responses. Conversely, participants who lacked a BI effect should generate a similar number of CBA and ABA responses.

### Materials and Procedure

#### Apparatus

**FIGURE 3:**
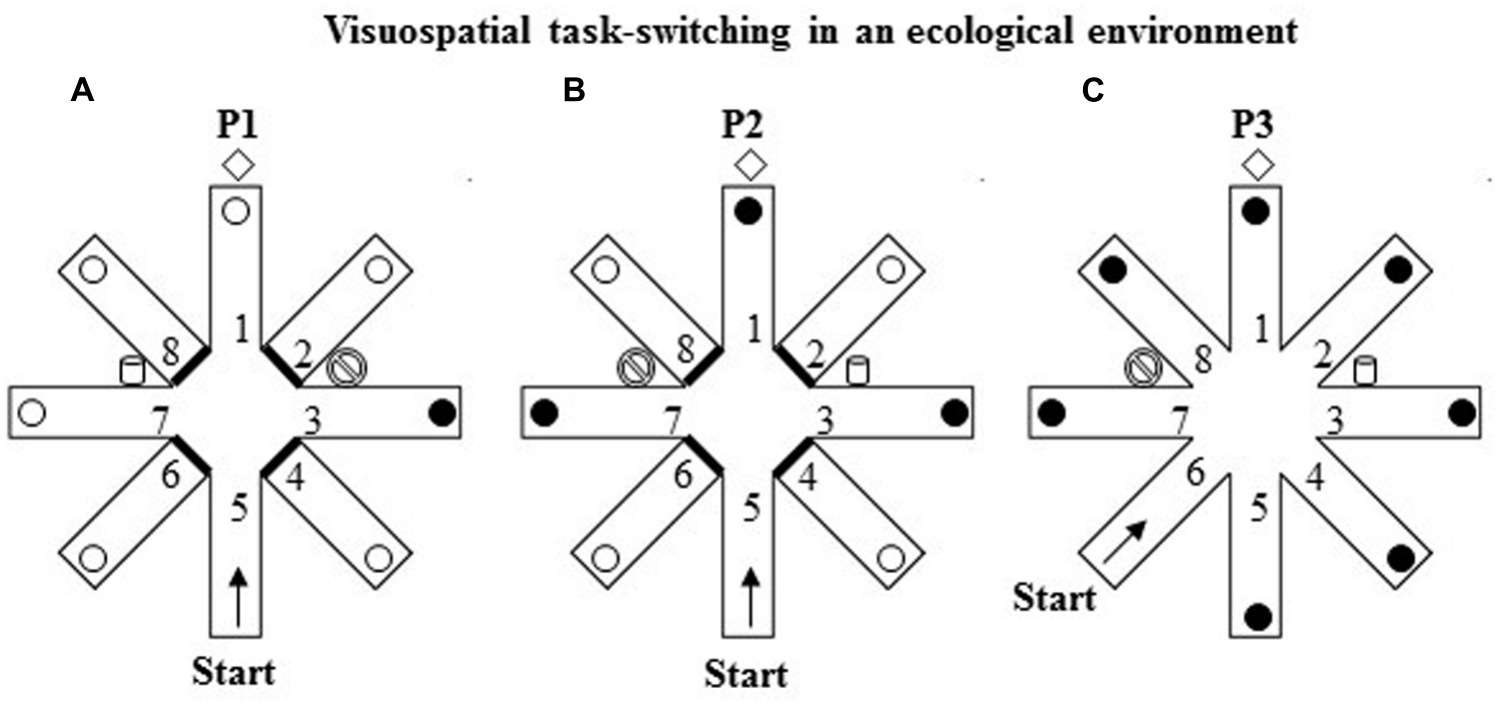
**Schematic representation of the VsTS paradigm in an ecological environment (Experiment 2).** **(A)** In the first phase (P1), the RAM had three intra-maze cues placed at the proximal ends of arms 3 and 7, and at the distal end of arm 1. The proximal ends of the arms 2, 4, 6, and 8 were closed. The participant started from the arm 5 and had to search for a reward put always in arm 3 until the criterion of three consecutive visits in the rewarded arm was reached. **(B)** In the second phase (P2), consisting of one trial, the cues of arms 3 and 7 were switched; arms 1, 3, 7 were reinforced. **(C)** In the third phase (P3), consisting of one trial, all eight arms were opened. The participant started from the arm 6.

The RAM consisted of a round central platform (3 m in diameter) with eight arms (80 cm wide × 11 m long) radiating like the spokes of a wheel. White and red ribbons forming a sort of barrier marked off the sides of each arm to force the participants to return to the center of the starting platform before entering another arm and thus to prevent them from “cutting corners.” At the end of each arm, there was an orange plastic bucket (18 cm wide × 28 cm high) containing the reward (a plastic coin). The eight arms were virtually numbered as indicated in **Figure 3**. The RAM was located outdoors in a large square and was surrounded by extra-maze cues (trees, buildings, pavement, streetlamps, etc.) held in constant spatial relations throughout the experiment. Particular attention was paid to control the intra- and extra-maze environment in terms of cues, the location of the buckets, the position of the experimenter, and so on. Participants could see and access the maze only during the experimental sessions.

#### Experimental Procedure

The RAM procedure (Palermo et al., 2014a) includes three phases. In the first phase (P1), three evident intra-maze cues were placed in the RAM apparatus, two big plastic containers (52 cm wide × 64 cm high) one blue and one white placed at the proximal ends of arms 3 and 7, and a brown hall tree (35 cm wide × 180 cm high) placed at the distal end of arm 1. The proximal ends of the four arms 2, 4, 6, and 8 were closed. The participant started from the distal end of arm 5 and had to search for a reward unexpectedly put always in the same arm 3 until the criterion of three consecutive visits in the rewarded arm was reached (**Figure 3A**). After visiting an arm, regardless of the reward discovery, the participant was guided out of the maze and taken for 30 s to a place where he/she could not see the maze before starting another trial. During this interval, the bucket at the end of arm 3 was always reinforced.

In the second phase (P2; **Figure 3B**), consisting of only one trial, the intra-maze cues (the blue and white big buckets) of arms 3 and 7 were switched; arms 1, 3, 7 were reinforced without participants’ knowledge, while arms 2, 4, 6, and 8 were closed. The participant started from the distal end of arm 5.

In the third phase (P3; **Figure 3C**), consisting of only one trial, all eight arms were opened and the cues were kept as in P2. The participant started from the distal end of arm 6. Notably, in P2 and P3 tests all the open arms were rewarded without warning to avoid biasing one strategy in respect of possible others.

#### Parameters

In P1 we considered the *number of trials to criterion* to assess the performance level and the *number of ABA and CBA* sequences the participants generated to evaluate the BI effect. In P2 and P3, we recorded the *arm visited* to verify the tendencies to flexibly adapt navigational strategies in a changing environment.

### Results and Discussion

**FIGURE 4:**
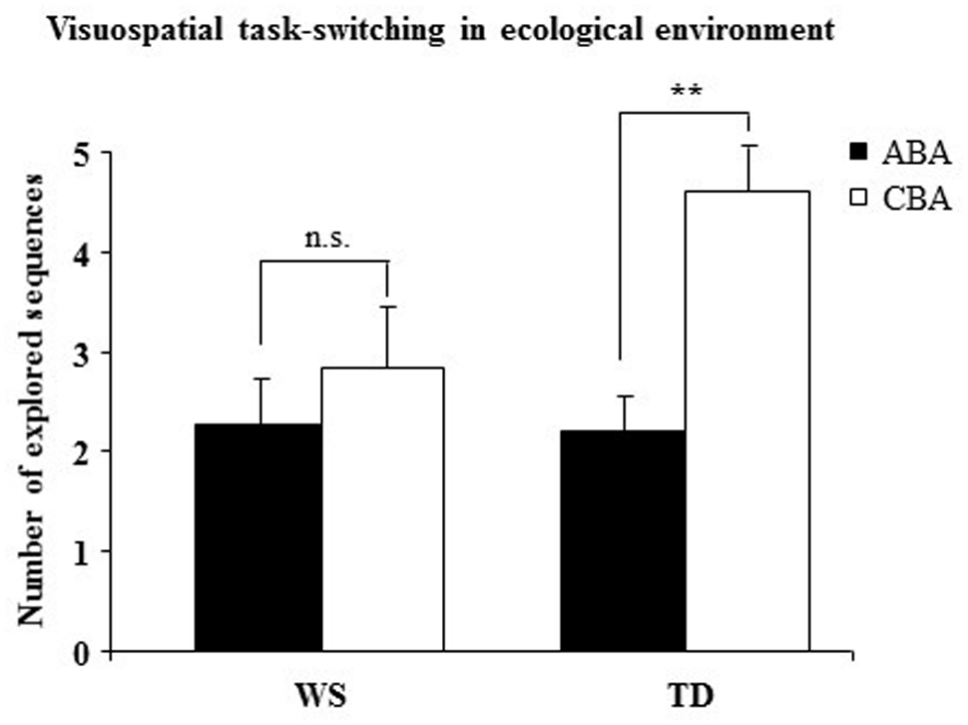
**Number of sequences of locations (ABA and CBA) that the participants spontaneously explored.** Data are expressed as mean ± SEM. The asterisks indicate the significance level of the *post hoc* comparisons between groups (^∗∗^*p*< 0.005).

In P1 the *number of trials to criterion* was similar between WS and TD groups (WS $\overset{-}{x}$ = 11.07 ± 1.38; TD $\overset{-}{x}$ = 11.20 ± 1.19), indicating a similar level of performance [*F*_(1,28)_ = 0.0013, *p* = 0.94, $\eta_{p}^{2}$ = 0.0002]. A two-way ANOVA (group × sequence) on the *number of CBA and ABA sequences* revealed a not significant group effect [*F*_(1,28)_ = 2.38, *p* = 0.13, $\eta_{p}^{2}$ = 0.08], whereas the sequence effect [*F*_(1,28)_ = 14.41, *p* = 0.0007, $\eta_{p}^{2}$ = 0.34] and interaction [*F*_(1,28)_ = 5.45, *p* = 0.02, $\eta_{p}^{2}$ = 0.16] were significant. *Post-hoc* comparisons on interaction revealed that while TD participants generated significantly more CBA than ABA sequences (*p* = 0.001)*,* WS participants generated almost the same number of CBA and ABA sequences (*p* = 0.31; **Figure 4**).

In P2, in spite of the changes of intra-maze cues, 80% of WS participants continued to choose the previously visited arm (arm 3). This persistent choice was exhibited by only the 31.25% of TD sample.

In P3, in spite of the changes of the starting arm, the 40% of WS participants continued to choose the previously visited arm (arm 3). This persistent choice was exhibited by only the 6.6% (i.e., only one participant) of TD sample.

The lacking BI effect in WS individuals in Experiment 1 was confirmed by the results of this experiment. Namely, WS participants spontaneously generated a similar number of ABA and CBA sequences, indicating that their navigational strategies were not modulated by an inhibitory control. Conversely, TD children generated fewer ABA than CBA responses, a clear indication that they tended not to return to previously visited (hence, inhibited) arms. As in Experiment 1, no between-group difference in performance level was observed, as evidenced by the similar number of trials to criterion. In the presence of changes of salient intra-maze cues (P2) or of starting arm (P3), WS individuals displayed an explorative rigidity indicated by their persistent choice of the previously visited arm. The low percentages of TD children who exhibited such an explorative pattern could indicate that they reacted to a changing context more flexibly and adaptively. Notably, P2 and P3 results demonstrate once again WS failure to adapt search strategies to previous outcomes and the propensity to emit fixed responses even in the presence of salient environmental changes. The rigid explorative pattern of WS individuals might be due to a deficient inhibitory control that did not permit to inhibit the already visited locations.

It remained to be verified whether the lack of BI in WS participants is limited to spatial domain or if it is part of a more general deficit. To address this issue, we tested the same groups of participants in a VeTS.

## Experiment 3: Verbal Task-Switching (VeTS)

Verbal task-switching paradigm required participants to process verbal stimuli (names of familiar animals) without tapping any spatial component.

### Materials and Procedure

**FIGURE 5:**
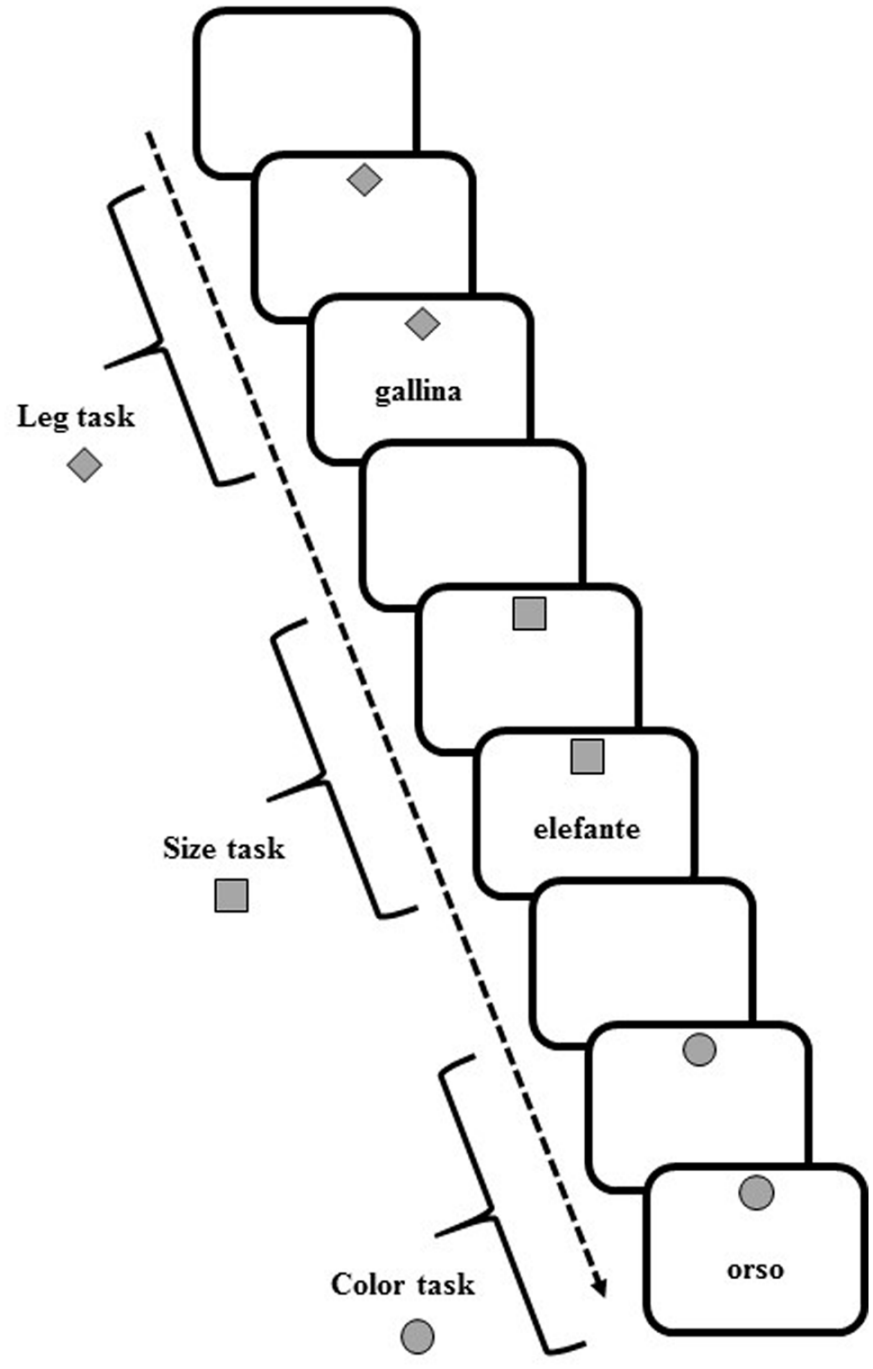
**Schematic representation of task cues and stimuli in the verbal task-switching (VeTS) paradigm (Experiment 3)**.

Participants sat in front of a computer screen, on which the target and cue stimuli appeared. White or gray (with equal probability) target stimuli (7^∘^width × 7^∘^height visual angle) comprised words of animals (elephant, bear, ostrich, chimpanzee, turtle, rabbit, chick, rooster) that appeared at the center on the screen. At the top of the screen, a gray cue (7^∘^width × 7^∘^height of visual angle) in various shapes (square, diamond, and circle) appeared on a white background (**Figure 5**).

In each trial, participants were asked to perform one of three tasks with the current stimulus, determining: (a) the number of legs of the animal (2-footed or 4-footed); (b) the actual size of the animal (small or large); and (c) the color of the writing of the stimulus (white or gray).

The upcoming task was pre-cued by the diamond to indicate an upcoming “leg number” task, the square for the “size” task, or the circle for the “color” task. In each trial, the cue appeared for 1000 ms and was followed by the target stimulus. For all tasks, the cue and target stimuli remained on the screen until a response was given or 10,000 ms had elapsed. For all trials, the response was followed by white screen for 700 ms, after which the next cue appeared.

Participants pressed one of two response buttons (the “A” and “L” keys) on a computer keyboard with the left and right index finger, respectively. Participants responded to the 2-footed, small, or white-sketched animal with the left hand and to the 4-footed, large, or gray-sketched animal with the right hand. Each participant underwent 189 trials in which randomized series of non-alternating and alternating ABA three-trial sequences (triplets) appeared. In a CBA sequence, three different tasks were executed (legs-color-size, size-color-legs, etc.); in an alternating ABA sequence, the same task was performed for the first and third trial (legs-color-legs, size-color-size, etc.). Twenty-four alternating and 27 non-alternating sequences were presented. Given the randomized presentation and lack of interval between triplets, participants were unaware that different sequences were presented.

#### Parameters

In VeST the *percentage of correct responses* was computed. To determine BI effect, the *RTs* in the third trials of ABA sequences were compared with RTs in the third trials of the CBA sequences. Only triplets for which participants responded correctly to all trials were used to compute BI effect.

### Results and Discussion

**FIGURE 6:**
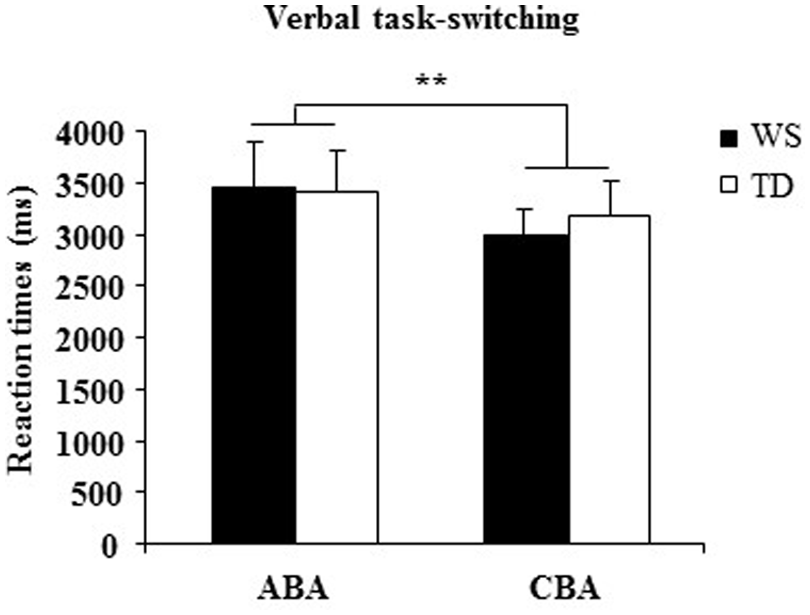
**Reaction times (RTs) in ABA and CBA sequences of trials for WS and TD participants.** Data are expressed as mean ± SEM. The asterisks indicate the significance level of sequence effect (^∗∗^*p*= 0.005).

A one-way ANOVA on the *percentage of correct responses* of WS and TD groups showed that WS individuals responded less accurately (_$\overset{-}{x}$_=69.50± 1.85) than TD participants [_$\overset{-}{x}$_=91.14± 1.79; *F*_(1,28)_ = 70.33, *p* < 0.00001, $\eta_{p}^{2}$ = 0.72]. A two-way ANOVA (group × sequence) on *RTs* on ABA and CBA sequences did not reveal a significant group effect [*F*_(1,28)_ = 0.022, *p* = 0.88, $\eta_{p}^{2}$ = 0.0008] and interaction [*F*_(1,28)_ = 1.01, *p* = 0.32, $\eta_{p}^{2}$ = 0.03], whereas sequence effect was significant [*F*_(1,28)_ = 9.07, *p* = 0.005, $\eta_{p}^{2}$ = 0.24; **Figure 6**].

The main result of Experiment 3 is the significant increase in RTs in both groups when they switched to a task set that had been abandoned two trials earlier (ABA sequences). This effect is consistent with the presence of an inhibitory process that alters the cognitive configurations to be abandoned. Interestingly, in WS participants the BI effect was lacking when visuospatial stimuli had to be processed and occurred as in TD children when verbal stimuli had be processed. The impaired visuospatial BI and preserved verbal BI in WS individuals is a clear demonstration of specific anomalies in their inhibitory control heavily involved also in processing of spatial information.

## General Discussion

This study was aimed at examining whether WS deficits in navigational abilities are related to eventual alterations of the spatial BI. In the VsTS paradigms of Experiments 1 and 2, the WS individuals failed to adapt explorative strategies to the previous outcomes and they adopted inflexible navigational strategies. Conversely, TD children tended not to return to previously visited (hence, inhibited) locations and flexibly reacted to the changing context. These findings suggest that WS navigational strategies were not modulated by the inhibitory control provided by the BI. Interestingly, in the VeTS paradigm of Experiment 3 both WS and TD participants showed a clear BI effect. Furthermore, WS individuals when tested in Experiment 2 showed atypical processing of both allocentric and egocentric spatial representations in accordance with the results of previous studies (Nardini et al., 2008; Farran et al., 2010; Foti et al., 2011; Bernardino et al., 2013; Broadbent et al., 2014). The present findings emphasize that the WS explorative strategies are not only inefficient, as suggested by Broadbent et al. (2014), but also inflexible. The ability to update the location of the self during navigation within an allocentric frame of reference is based on updating and switching between egocentric and allocentric spatial codes (Burgess, 2008). An efficient BI permits these processes to be efficiently executed. Thus, an exhaustive interpretation of the WS navigational deficits has to take into account BI involvement in the exploration. In this regard, the WS difficulties in inhibitory processes have been described (Carney et al., 2013). Specifically, WS individuals fail to withhold a response and re-engage attentional control after an error (Greer et al., 2013). Moreover, they show deficits in some executive functions (as selective and sustained attention, short-term memory, planning) in both verbal and visuospatial modalities, but their shifting abilities are impaired only in the visuospatial tasks (Menghini et al., 2010; Rhodes et al., 2010, 2011).

Why the BI could be important for an efficient navigation? Typically, the BI reduces interference originating from previously stored information and facilitates the instantiation of new information, allowing individuals to adapt flexibly to contexts (Mayr and Keele, 2000; Koch et al., 2010). To successfully navigate, it is necessary to activate the general and schematic representation of the environment (cognitive map), “zooming” then on the sector in which the individual is actually moving. While the general representation is kept stable, the zoom is continuously shifted to update it according to individual’s translations. The BI may support this process by reducing the activation level of the previous representation in the spatial working memory buffer (Sdoia and Ferlazzo, 2012). A crucial requisite of any memory system is the maintenance in working memory of the active representation of stored information, avoiding at the same time it may interfere with the formation of new representations. Thus, BI role in navigation may be not to allow previous representations to interfere with the next ones.

Additionally, even the other facet of BI function related to switching among representations may be functional to a successful navigation that requires developing multiple spatial representations. Space can be subdivided into peripersonal and extrapersonal as a function of spatial position of the individual (Previc, 1998), resulting in a dissociation between egocentric and allocentric space. The visuospatial system is forced to switch continually between egocentric and allocentric systems to cope with the multiple demands of complex environment (Igloi et al., 2009; Wolbers and Hegarty, 2010). Thus, BI process mediating such an interchangeable shifting is required to efficiently explore.

Were this the case, WS individuals could have navigational difficulties whenever a competition among items in the spatial working memory buffer occurs, while they could find their way whenever the representation needed to drive navigation does not require switching among zoomed representations of the environment. In other words, the daily navigational difficulties of WS individuals could be linked to an impaired updating of environmental representations during navigation because of their altered visuospatial BI.

## Author Contributions

All authors designed research; FF and SS tested participants; all authors analyzed data and discussed data; FF, SS, FF, and LP wrote the paper. All authors read, revised, and approved the final manuscript.

## Conflict of Interest Statement

The authors declare that the research was conducted in the absence of any commercial or financial relationships that could be construed as a potential conflict of interest.

## References

[B1] Arbuthnott K. (2008). The effect of task location and task type on backward inhibition. *Mem. Cogn.* 36 534–543 10.3758/MC.36.3.534 18491493

[B2] Atkinson J. Braddick O. (2011). From genes to brain development to phenotypic behavior: ‘dorsal-stream vulnerability’ in relation to spatial cognition, attention, and planning of actions in Williams syndrome (WS) and other developmental disorders. *Prog. Brain Res.* 189 261–283 10.1016/B978-0-444-53884-0.00029-4 21489394

[B3] Atkinson J. Braddick O. Anker S. Curran W. Andrew R. Wattam-Bell J. (2003). Neurobiological models of visuospatial cognition in children with Williams syndrome: measures of dorsal-stream and frontal function. *Dev. Neuropsychol.* 23 139–172 10.1080/87565641.2003.9651890 12730023

[B4] Beery K. E. Buktenica N. A. (2000). *The Beery–Buktenica Developmental Test of Visual-Motor Integration with Additional Tests of Visual Perception and Motor Coordination [Italian translation by C. Preda]*. Florence: O.S. Organizzazioni Speciali.

[B5] Bellugi U. Lichtenberger L. Jones W. Lai Z. St George M. I. (2000). The neurocognitive profile of Williams syndrome: a complex pattern of strengths and weaknesses. *J. Cogn. Neurosci.* 12 7–29 10.1162/089892900561959 10953231

[B6] Bernardino I. Mouga S. Castelo-Branco M. van Asselen M. (2013). Egocentric and allocentric spatial representations in Williams syndrome. *J. Int. Neuropsychol. Soc.* 19 54–62 10.1017/S1355617712000963 23095237

[B7] Bianchini F. Incoccia C. Palermo L. Piccardi L. Zompanti L. Sabatini U. (2010). Developmental topographical disorientation in a healthy subject. *Neuropsychologia* 48 1563–1573 10.1016/j.neuropsychologia.2010.01.025 20144632

[B8] Bianchini F. Palermo L. Piccardi L. Incoccia C. Nemmi F. Sabatini U. (2014). Where am I? A new case of developmental topographical disorientation. *J. Neuropsychol.* 8 107–124 10.1111/jnp.12007 23336564

[B9] Broadbent H. J. Farran E. K. Tolmie A. (2014). Egocentric and allocentric navigation strategies in Williams syndrome and typical development. *Dev. Sci.* 17 920–934 10.1111/desc.12176 24702907

[B10] Brock J. (2007). Language abilities in Williams syndrome: a critical review. *Dev. Psychopathol.* 19 97–127 10.1017/S095457940707006X 17241486

[B11] Burgess N. (2006). Spatial memory: how egocentric and allocentric combine. *Trends. Cogn. Sci.* 10 551–557 10.1016/j.tics.2006.10.005 17071127

[B12] Burgess N. (2008). Spatial cognition and the brain. *Ann. N. Y. Acad. Sci.* 1124 77–97 10.1196/annals.1440.002 18400925

[B13] Carney D. P. Brown J. H. Henry L. A. (2013). Executive function in Williams and downsyndromes. *Res. Dev. Disabil.* 34 46–55 10.1016/j.ridd.2012.07.013 22940158

[B14] Costanzo F. Varuzza C. Menghini D. Addona F. Gianesini T. Vicari S. (2013). Executive functions in intellectual disabilities: a comparison between Williams syndrome and Down syndrome. *Res. Dev. Disabil.* 34 1770–1780 10.1016/j.ridd.2013.01.024 23501586

[B15] Dagenbach D. Carr T. H. Menzer D. Duquette P. J. Chalk H. M. Rupard M. (2007). “Adventures in inhibition: plausibly, but not certifiably, inhibitory processes,” in *Inhibition in Cognition*, eds Gorfein D. S. MacLeod C. M. (Washington, DC: American Psychological Association).

[B16] Ewart A. K. Morris C. A. Atkinson D. Jin W. Sternes K. Spallone P. (1993). Hemizygosity at the elastin locus in a developmental disorder, Williams syndrome. *Nat. Genet.* 5 11–16 10.1038/ng0993-11 7693128

[B17] Farran E. K. Blades M. Boucher J. Tranter L. J. Jarrold C. Stinton C. (2010). How do individuals with Williams syndrome learn a route in a real-world environment? *Dev*. *Sci.* 13 454–468 10.1111/j.1467-7687.2009.00894.x 20443966

[B18] Farran E. K. Courbois Y. Van Herwegen J. Blades M. (2012). How useful are landmarks when learning a route in a virtual environment? Evidence from typical development and Williams syndrome. *J. Exp. Child Psychol.* 111 571–586 10.1016/j.jecp.2011.10.009 22244218

[B19] Farran E. K. Jarrold C. (2004). Exploring block construction and mental imagery: evidence of atypical orientation discrimination in Williams syndrome. *Vis. Cogn.* 11 1019–1039 10.1080/13506280444000058b

[B20] Farran E. K. Jarrold C. Gathercole S. E. (2001). Block design performance in the Williams syndrome phenotype: a problem with mental imagery? *J*.*Child Psychol. Psychiatry* 42 719–728 10.1111/1469-7610.00768 11583244

[B21] Foti F. Petrosini L. Cutuli D. Menghini D. Chiarotti F. Vicari S. (2011). Explorative function in Williams syndrome analyzed through a large-scale task with multiple reward. *Res. Dev. Disabil.* 32 972–985 10.1016/j.ridd.2011.02.001 21353462

[B22] Greer J. Riby D. M. Hamiliton C. Riby L. M. (2013). Attentional lapse and inhibition control in adults with Williams Syndrome. *Res. Dev. Disabil.* 34 4170–4177 10.1016/j.ridd.2013.08.041 24076981

[B23] Hocking D. R. Bradshaw J. L. Rinehart N. J. (2008). Fronto-parietal and cerebellar contributions to motor dysfunction in Williams syndrome: a review and future directions. *Neurosci. Biobehav. Rev.* 32 497–507 10.1016/j.neubiorev.2007.09.003 18006060

[B24] Hoffman J. E. Landau B. Pagani B. (2003). Spatial breakdown in spatial construction: evidence from eye fixations in children with Williams syndrome. *Cogn. Psychol.* 46 260–301 10.1016/S0010-0285(02)00518-2 12694695

[B25] Iaria G. Bogod N. Fox C. J. Barton J. J. (2009). Developmental topographical disorientation: case one. *Neuropsychologia* 47 30–40 10.1016/j.neuropsychologia.2008.08.021 18793658

[B26] Iaria G. Incoccia C. Piccardi L. Nico D. Sabatini U. Guariglia C. (2005). Lack of orientation due to a congenital brain malformation: a case study. *Neurocase* 11 463–474 10.1080/13554790500423602 16393760

[B27] Igloi K. Zaoui M. Berthoz A. Rondi-Reig L. (2009). Sequential egocentric strategy is acquired as early as allocentric strategy: parallel acquisition of these two navigation strategies. *Hippocampus* 19 1199–1211 10.1002/hipo.20595 19360853

[B28] Jarrold C. Baddeley A. D. Phillips C. (2007). Long-term memory for verbal and visual information in Down syndrome and Williams syndrome: performance on the doors and people test. *Cortex* 43 233–247 10.1016/S0010-9452(08)70478-7 17405669

[B29] Karmiloff-Smith A. (2012). Perspectives on the dynamic development of cognitive capacities: insights from Williams syndrome. *Curr. Opin. Neurol.* 25 106–111 10.1097/WCO.0b013e3283518130 22322417

[B30] Kiesel A. Steinhauser M. Wendt M. Falkenstein M. Jost K. Philipp A. M. (2010). Control and interference in task switching–a review. *Psychol. Bull.* 136 849–874 10.1037/a0019842 20804238

[B31] Koch I. Gade M. Schuch S. Philipp A. M. (2010). The role of inhibition in task switching: a review. *Psychon. Bull. Rev.* 17 1–14 10.3758/PBR.17.1.1 20081154

[B32] Kozhevnikov M. Motes M. A. Rasch B. Blajenkova O. (2006). Perspective-taking vs. mental rotation transformations and how they predict spatial navigation performance. *Appl. Cogn. Psychol.* 20 397–417 10.1002/acp.1192

[B33] Leyfer O. T. Woodruff-Borden J. Klein-Tasman B. P. Fricke J. S. Mervis C. B. (2006). Prevalence of psychiatric disorders in 4 to 16-year-olds with Williams syndrome. *Am. J. Med. Genet. B Neuropsychiatr. Genet*. 141 615–622 10.1002/ajmg.b.30344 16823805 PMC2561212

[B34] Mandolesi L. Addona F. Foti F. Menghini D. Petrosini L. Vicari S. (2009). Spatial competences in Williams syndrome: a radial arm maze study. *Int. J. Dev. Neurosci.* 27 205–213 10.1016/j.ijdevneu.2009.01.004 19429385

[B35] Martens M. A. Wilson S. J. Reutens D. C. (2008). Research review: Williams syndrome: acritical review of the cognitive, behavioral, and neuroanatomical phenotype. *J. Child. Psychol. Psychiatry* 49 576–608 10.1111/j.1469-7610.2008.01887.x 18489677

[B36] Mayr U. Keele S. W. (2000). Changing internal constraints on action: the role of backward inhibition. *J. Exp. Psychol. Gen.* 129 4–26 10.1037/0096-3445.129.1.4 10756484

[B37] Menghini D. Addona F. Costanzo F. Vicari S. (2010). Executive functions in individuals with Williams syndrome. *J. Intellect. Disabil. Res.* 54 418–432 10.1111/j.1365-2788.2010.01287.x 20537048

[B38] Meyer-Lindenberg A. Mervis C. B. Sarpal D. Koch P. Steele S. Kohn P. (2005). Functional, structural, and metabolic abnormalities of the hippocampal formation in Williams syndrome. *J. Clin. Invest.* 115 1888–1895 10.1172/jci24892 15951840 PMC1143592

[B39] Miyake A. Friedman N. P. (2012). The Nature and organization of individual differences in executive functions: four general conclusions. *Curr. Dir. Psychol. Sci.* 21 8–14 10.1177/0963721411429458 22773897 PMC3388901

[B40] Miyake A. Friedman N. P. Emerson M. J. Witzki A. H. Howerter A. Wager T. D. (2000). The unity and diversity of executive functions and their contributions to complex “Frontal Lobe” tasks: a latent variable analysis. *Cogn. Psychol.* 41 49–100 10.1006/cogp.1999.0734 10945922

[B41] Miyake A. Shah P. (1999). Models of Working Memory: Mechanisms of Active Maintenance and Executive Control. New York, NY: Cambridge University Press. 10.1017/CBO9781139174909

[B42] Nardini M. Atkinson J. Braddick O. Burgess N. (2008). Developmental trajectories for spatial frames of reference in Williams syndrome. *Dev. Sci.* 11 583–595 10.1111/j.1467-7687.2007.00662 18576966

[B43] Palermo L. Foti F. Ferlazzo F. Guariglia C. Petrosini L. (2014a). I find my way in a maze but not in my own territory! navigational processing in developmental topographical disorientation. *Neuropsychology* 28 135–146 10.1037/neu0000021 24219605

[B44] Palermo L. Piccardi L. Bianchini F. Nemmi F. Giorgio V. Incoccia C. (2014b). Looking for the compass in a case of developmental topographical disorientation: a behavioral and neuroimaging study. *J. Clin. Exp. Neuropsychol.* 36 464–481 10.1080/13803395.2014.904843 24742227

[B45] Previc F. H. (1998). The neuropsychology of 3-D space. *Psychol. Bull*. 124 123–164 10.1037/0033-2909.124.2.123 9747184

[B46] Rhodes S. M. Riby D. M. Fraser E. Campbell L. E. (2011). The extent of working memory deficits associated with Williams syndrome: exploration of verbal and spatial domains and executively controlled processes. *Brain Cogn.* 77 208–214 10.1016/j.bandc.2011.08.009 21889249

[B47] Rhodes S. M. Riby D. M. Park J. Fraser E. Campbell L. E. (2010). Executive neuropsychological functioning in individuals with Williams syndrome. *Neuropsychologia* 48 1216–1226 10.1016/j.neuropsychologia.2009.12.021 20026085

[B48] Roid G. H. Miller L. J. (2002). Leiter–R, Leiter International Performance Scale–Revised. Firenze: Giunti O.S. Organizzazioni Speciali.

[B49] Sdoia S. Ferlazzo F. (2012). An inhibition effect in the temporal constrains of attentional selection: the backward blink. *Acta Psychol.* 139 501–506 10.1016/j.actpsy.2012.01.005 22365900

[B50] Siegel A. W. White S. H. (1975). “The development of spatial representations of large-scale environments,” in *Advances in Child Development and Behavior* Vol. 10 ed. Reese H. W. (New York, NY: Academic Press), 531–549. 10.1016/s0065-2407(08)60007-5 1101663

[B51] Stinton C. Farran E. K. Courbois Y. (2008). Mental rotation in Williams syndrome: an impaired ability. *Dev. Neuropsychol.* 33 565–583 10.1080/87565640802254323 18788011

[B52] Stromme P. Bjomstad P. G. Ramstad K. (2002). Prevalence estimation of Williams syndrome. *J. Child Neurol.* 17 269–271 10.1177/088307380201700406 12088082

[B53] Towse J. N. Neil D. (1998). Analyzing human random generation behavior: a review of methods used and a computer program for describing performance. *Behav. Res. Methods Instrum. Comput.* 30 583–591 10.3758/BF03209475

[B54] Vandierendonck A. Liefooghe B. Verbruggen F. (2010). Task switching: interplay of reconfiguration and interference control. *Psychol. Bull.* 136 601–626 10.1037/a0019791 20565170

[B55] Vicari S. (2007). PROMEA: Prove di Memoria e Apprendimento. Florence: Giunti – Organizzazioni Speciali.

[B56] Vicari S. Bellucci S. Carlesimo G. A. (2005). Visual and spatial long-term memory: differential pattern of impairments in Williams and down syndromes. *Dev. Med. Child Neurol*. 47 305–311 10.1111/j.1469-8749.2005.tb01141.x 15892372

[B57] Vicari S. Bellucci S. Carlesimo G. A. (2006). Evidence from two genetic syndromes for the independence of spatial and visual working memory. *Dev. Med. Child Neurol*. 48 126–131 10.1017/S0012162206000272 16417668

[B58] Wolbers T. Hegarty M. (2010). What determines our navigational abilities? *Trends Cogn. Sci.* 14 138–146 10.1016/j.tics.2010.01.001 20138795

